# A Novel Pathogenesis-Related Class 10 Protein *Gly m 4l,* Increases Resistance upon *Phytophthora sojae* Infection in Soybean (*Glycine max* [L.] Merr.)

**DOI:** 10.1371/journal.pone.0140364

**Published:** 2015-10-16

**Authors:** Sujie Fan, Liangyu Jiang, Junjiang Wu, Lidong Dong, Qun Cheng, Pengfei Xu, Shuzhen Zhang

**Affiliations:** 1 Soybean Research Institute, Key Laboratory of Soybean Biology of Chinese Education Ministry, Northeast Agricultural University, Harbin, 150030, Heilongjiang, People’s Republic of China; 2 Soybean Research Institute, Heilongjiang Academy of Agricultural Sciences, Collaborative Innovation Center of Grain Production Capacity Improvement in Heilongjiang Province, Harbin, 150086, Heilongjiang, People’s Republic of China; Nanjing Agricultural University, CHINA

## Abstract

Phytophthora root and stem rot of soybean, caused by *Phytophthora sojae* (*P*. *sojae*), is a destructive disease in many soybean planting regions worldwide. In a previous study, an expressed sequence tag (EST) homolog of the major allergen *Pru ar 1* in apricot (*Prunus armeniaca*) was identified up-regulated in the highly resistant soybean ‘Suinong 10’ infected with *P*. *sojae*. Here, the full length of the EST was isolated using rapid amplification of cDNA ends (RACE). It showed the highest homolgy of 53.46% with *Gly m 4* after comparison with the eight soybean allergen families reported and was named *Gly m 4-like* (*Gly m 4l*, GenBank accession no. HQ913577.1). The cDNA full length of *Gly m 4l* was 707 bp containing a 474 bp open reading frame encoding a polypeptide of 157 amino acids. Sequence analysis suggests that *Gly m 4l* contains a conserved ‘P-loop’ (phosphate-binding loop) motif at residues 47–55 aa and a Bet v 1 domain at residues 87–120 aa. The transcript abundance of *Gly m 4l* was significantly induced by *P*. *sojae*, salicylic acid (SA), NaCl, and also responded to methyl jasmonic acid (MeJA) and ethylene (ET). The recombinant *Gly m 4l* protein showed RNase activity and displayed directly antimicrobial activity that inhibited hyphal growth and reduced zoospore release in *P*. *sojae*. Further analyses showed that the RNase activity of the recombinant protein to degrading tRNA was significantly affected in the presence of zeatin. Over-expression of *Gly m 4l* in susceptible ‘Dongnong 50’ soybean showed enhanced resistance to *P*. *sojae*. These results indicated that *Gly m 4l* protein played an important role in the defense of soybean against *P*. *sojae* infection.

## Introduction

Pathogenesis-related (PR) proteins were initially described as a class of plant-specific proteins that accumulated in response to pathogen infection; however, subsequent studies showed that these proteins are induced through a variety of biotic and abiotic stresses [1–2]. In addition, PR proteins are reported constitutively expressed in different tissues and organs during growth [3–4] and are currently classified into 17 functional families based on primary structure, serological relationships, and biological activities [1, 4]. Most PR proteins are localized in the vacuole extracellularly or intracellularly. In contrast, PR10 proteins are intracellular and cytoplasmic [5].

Most members of the PR10 protein contain an open reading frame (ORF) of 456 to 696 nucleotides, usually interrupted by an intron of 76–359 nucleotides, which encode a polypeptide of 151–231 amino acids with predicted molecular masses of 15–26 kDa [6–8]. In general, the 3D structures of PR10 proteins contain a 25-amino-acid long α-helix (α3) in C-terminal and two short α-helices (α1 and a2) in N-terminal. The α3 is surrounded by seven anti-parallel β-sheets (β1 to β7) and the α1 and α2 are located between β1 and β2 strands [9–10].

The PR10 proteins, first described in cultured parsley (*Petroselinum crispum*) cells [11], have been reported in numerous plant species when induced by various pathogens such as bacteria, fungi, oomycetes, and viruses [12–20]. Moreover, PR10 genes are also induced through various abiotic stresses, such as salt, heat, cold, PEG, dark, drought, ozone, UV irradiation, copper, H_2_O_2_ and wounding [6, 18, 21–29]. The expression levels of some PR10 genes are regulated following treatments with signaling molecules and plant hormones [30–32].

PR10 proteins are not only involved in plant defense, they also play a role in developmental processes [33] and exhibit enzymatic activities in secondary metabolism [34], suggesting that these proteins constitute a multifunctional protein family [35–36]. The structural similarity between PR10 proteins and ribonucleases from phosphate-starved ginseng (*Panax ginseng*) cells suggests that PR10 proteins function as ribonucleases [37], and several studies have shown that members of the PR10 protein family possess RNA degrading activities [38–39]. Notably, Park et al.[20] showed that CaPR10 from hot pepper (*Capsicum annuum*) exhibited RNase activity and also demonstrated that this protein was directly involved in antiviral and antimicrobial processes *in vitro*. Another study demonstrated that the ribonuclease activity of AhPR10 from peanut (*Arachis hypogaea* L.) was essential for the antifungal activity of this protein, as the loss of the ribonuclease in mutants resulted in a loss of antifungal activity [5]. In addition, the RNase and DNase activities of PBZ1, a PR10 family protein in rice (*Oryza sativa*), and VpPR-10.1, from Chinese wild grape (*Vitis pseudoreticulata*), have been shown to induce programmed cell death and DNA degradation [16, 40]. Most PR10 proteins have also been shown to bind to plant hormones, including cytokinins [41–42], brassinosteroids [10], and hydrophobic ligands [43], which might be important for plant defense responses to pathogens and plant growth and development [10].

In recent years, a variety of PR proteins and homologs causing allergenicity in humans have been isolated and characterized. Bet v 1, belonging to PR10 family, isolated from *Betula verrucosa*, is the main allergen present in pollen grains [44]. Subsequently, a considerable number of diverse plant allergen proteins with sequence similarity to Bet v 1 have been identified, such as Mal d 1 from *Malus domestica* [45], Pru ar 1 from *Prunus armeniaca* (AF020784), Pru av 1 from *Prunus avium* [46], Pyr c 1 from *Pyrus communis* (AF057030), Dau c 1 from *Daucus carota* [47], Pru p 1 from *Prunus persica* [48] and Api g 1 from *Apium graveolens* [49]. In soybean, a variety of allergens have been identified into eight families (http://www.allergen.org) till now, namely Gly m 1 [50], Gly m 2 [51], Gly m 3 [52], Gly m 4 [53–55], Gly m 5 [56–57], Gly m 6 [56–57], Gly m 7 and Gly m 8 [58]. Gly m 4, a member of the superfamily of Bet v 1 homologous proteins, are particularly similar to the PR10 proteins of yellow lupine [53–55].

In a previous study, we identified a novel up-regulated sequence tag (EST), homologous to major allergen Pru ar 1 from apricot (*Prunus armeniaca*) (GenBank Accession no. AF020784), in the highly resistant soybean ‘Suinong 10’ after infection with the oomycete *Phytophthora sojae* (*P*. *sojae*) [59]. In the present study, the full length of the EST was isolated using rapid amplification of cDNA ends (RACE). It showed identifies of 53.46% homology with Gly m 4 protein from *Glycine max*, representing the highest homology among the eight soybean allergen families, and was designated *Gly m 4-like* (*Gly m 4l*). The expression patterns of *Gly m 4l* induced under abiotic and biotic stresses were examined. The RNase activity of Gly m 4l protein and the influence of cytokinins on the RNase activity were established. Then, the antimicrobial activity of Gly m 4l protein against hyphal growth and zoospore release in *P*. *sojae* were investigated. Moreover, *in vivo*, over-expressing *Gly m 4l* gene in soybean plants under the control of 35S promoter were produced and the antimicrobial activity of transgenic plants were also investigated.

## Materials and Methods

### Plant Materials and Growth Conditions

‘Suinong 10’, a soybean cultivar with high resistance against the predominant race 1 of *P*. *sojae* in Heilongjiang, China [60], was used for gene isolation. *P*. *sojae* race 1, PSR01, which was isolated from infected soybean plants in Heilongjiang [60], was cultivated at 25°C for 7 days on V8 juice agar in a polystyrene dish.

‘Suinong 10’ soybean seeds were planted in pots filled with sterile vermiculite in a growth chamber with a 14 h photoperiod (at a light intensity of 350 mol.m^-2^s^-1^) at 22°C/ 18°C day/night temperatures and relative humidity of 90 ± 10%.

The seeds of ‘Dongnong 50’ soybean, susceptible to *P*. *sojae* race 1, were obtained from the Key Laboratory of Soybean Biology in Chinese Ministry of Education, Harbin and used for the gene transformation experiment.

### Isolation of the Full-Length *Gly m 4l* cDNA by 5′- and 3′- RACE and Sequence Analysis

In a previous study, a cDNA library of mRNAs encoding expressed sequence tags (ESTs) that showing increased expression during *P*. *sojae* infection was constructed using suppression subtractive hybridization (SSH) from the leaf tissues of the highly resistant soybean ‘Suinong 10’. A soybean up-regulated EST homolog of the major allergen *Pru ar 1* gene in apricot (*Prunus armeniaca*) was identified using microarray analysis and real-time PCR [59]. In the present study, the full-length cDNA was isolated by way of rapid amplification of cDNA ends (RACE), performed using the SMART RACE cDNA amplification Kit (Clontech, CA, USA) according to the manufacturer’s instructions. The gene-specific primers GSP1 for 5′- RACE and GSP2 for 3′- RACE were designed to amplify the antisense and sense strands, respectively (see S1 Table for primer sequences). All RACE PCR reactions were performed at 94°C for 5 min, followed by 35 cycles at 94°C for 30 s, 57°C for 30 s, and 72°C for 2 min, with a final extension at 72°C for 10 min. The PCR products were cloned into pMD^TM^18-T vector (Promega, USA), then transformed into *E*. *coli* DH5α cells (Shanghai Biotech Inc, Shanghai, China) and sequenced (GENEWIZ, Beijing, China). After RACE, a single full-length cDNA sequence was obtained by combining the 5′- RACE fragment, the EST fragment and the 3′-RACE fragment. Then, the full-length cDNA was gotten through the amplification of the cDNA template reversed transcribed from total RNA using the primer pairs F and R (S1 Table). The full length genomic DNA was also amplified using the primers mentioned above. The amplification products were gel purified and cloned into pMD^TM^18-T vector and sequenced.

Splicing sequences were corrected with the online BLAST programs (http://www.phytozome.net/Glycine max; http://www.ncbi.nlm.nih.gov/BLAST). Sequence alignments of nucleotides and amino acids sequences were performed using DNAMAN software (http://www.lynnon.com/). The evolutionary tree of amino acid sequence of Gly m 4l and other PR10 members was designed to search for homologous proteins using MEGA 5.1 software (http://www.megasoftware.net). Three dimensional (3D) structure of Gly m 4l was predicted based on the online program Phyre 2 (http://www.sbg.bio.ic.ac.uk/phyre2).

### Application of Abiotic and Biotic Stresses

To investigate *Gly m 4l* gene expression profiling, the seedlings of ‘Suinong 10’ soybean at the first-node stage (V1) [61] were used for various treatments.

To examine the effect of exogenous chemicals and stresses on Gly m 4l expression, the ‘Suinong 10’ soybean seedlings were treated with 0.5 mM salicylic acid (SA), 100 μM methyl jasmonic acid (MeJA), 50 mM abscisic acid (ABA), 50 mg.L^-1^ gibberellic acid (GA_3_), 100 mM NaCl, or 20% polyethylene glycol (PEG) (Sigma-Aldrich, USA). The phytohormone was first dissolved in 0.1% (v/v) ethanol (hormone solvent) and subsequently diluted to a working concentration with deionized water. The controls were treated with the same dilutions without phytohormone. For treatment with ethylene (ET), ‘Suinong 10’ soybean seedlings were maintained in a chamber equilibrated with 5% (v/v) gaseous ethylene. The ethylene concentration was confirmed through gas chromatography. Control experiments were performed in an identical chamber without ethylene. After induction, the soybean seedlings were placed in the appropriate growth chamber with a 14 h photoperiod (at a light intensity of 350 mol. m^-2^s^-1^) at 22°C/18°C day/night temperatures. For cold induction, the soybean seedlings were maintained in a 4°C incubator, and the control experiments were performed in the growth chamber at 22°C/18°C day/night temperatures. The leaves of soybean seedlings were harvested for RNA isolation at 0, 1, 3, 6, 9, 12, and 24 h after treatments with SA, MeJA, ET, ABA and GA_3_. The leaves of soybean seedlings were harvested for RNA isolation at 0, 1, 6, 12, 24, 36, and 72 h after treatments with NaCl, PEG, and cold.

For *P*. *sojae* treatment, the soybean seedlings were infected with zoospores according to the method of Ward et al. (1979) [62]. After inoculation, the ‘Suinong 10’ soybean seedlings were kept at 90 ± 10% humidity conditions and the leaves were harvested at 0, 6, 12, 24, 36, and 72 h after the treatment, immediately frozen in liquid nitrogen, and stored at -80°C until RNA extraction and cDNA synthesis.

### qPCR Analysis

The expression profiling of *Gly m 4l* was determined through qPCR analyses, performed using a real-time RT-PCR kit according to the manufacturer’s instructions (Takara, Japan) on a CFX96 Touch^TM^ Real-Time PCR Detection System (Bio-Rad, USA). Total RNA was extracted using Trizol reagent (Invitrogen, Shanghai, China) following the manufacture’s protocol. The first-strand cDNA was obtained by approximately 1 μg of total RNA using M-MLV reverse transcriptase kit (Takara, Dalian, China). Each amplification reaction was performed in a total 20 μL reaction volume containing 10 μL of 2× SYBR Green PCR Master Mix, 150 nmoL of each specific primer *Gly m 4l*-qF or *Gly m 4l*-qR (S1 Table), and 10 ng of the synthesized cDNA. The PCR protocol included 95°C for 1 min followed 40 cycles at 95°C for 15 s, 60°C for 15 s, and 72°C for 45 s. The amplification product was confirmed through melting curve analysis from 95°C to 60°C every one degree. The relative expression value of different tissues was calculated by the 2^−ΔΔCt^ method using the soybean internal control gene *EF1β* (GenBank accession no. NM_001248778) with the primer pairs *GmEF1β*-F and *GmEF1β*-R (S1 Table). The relative expression value for treatments with abiotic and biotic stresses was calculated by the 2^−ΔΔCT^ method using the soybean internal control gene *GmActin 4* (GenBank accession no. AF049106) with the primer pairs *GmActin 4*-F and *GmActin 4*-R (S1 Table). The qPCR analyses were performed on three biological replicates using RNA samples extracted from three independent plants with their respective three technical replicates.

### Subcellular Localization of *Gly m 4l* with the Fusion of Green Fluorescent Protein (GFP)

The full-length coding region of *Gly m 4l* was cloned in frame into the 5’-terminus of the GFP coding sequence in 35S:GFP vector using the primer pairs *Gly m 4l*-lF and *Gly m 4l*-lR (S1 Table), generating the fusion construct 35S: Gly m 4l-GFP. The resulting fusion 35S: Gly m 4l-GFP or control 35S: GFP construct was introduced into Arabidopsis protoplasts via the polyethylene glycol (PEG) mediated transformation as described by Yoo et al. [63]. After incubation of transfected Arabidopsis protoplasts cells for 16 h at 25°C, the GFP signal was observed using a Confocal Laser Scanning Microscopy (Leica DMI 6000,Wetzlar, Germany).

### Expression and Purification of the Recombinant *Gly m 4l* Protein

The full-length coding region of *Gly m 4l* was inserted in frame into the pET-29b(+) vector (EMD Millipore, USA) to create a pET29b(+)-Gly m 4l. Subsequently, the plasmid pET-29b(+)-Gly m 4l was transformed into the *E*. *coli* BL21 (DE3) strain, and over-expression of the cloned genes was induced with 1 mM IPTG at 37°C for 5 h. For the recombinant protein purification, the bacterial cells were pelleted after induction, resuspended in 10 mL ice-cold 1×Binding Buffer (0.5 M NaCl, 20 mM Tris-HCl, 5 mM imidazole, pH 7.9), and sonicated on ice for 10 min (30 s pulse/min) until the sample was no longer viscous. Following centrifugation at 1200×g for 15 min at 4°C, the supernatant was harvested and loaded onto a His-bind Resin column (EMD Millipore, USA). The recombinant Gly m 4l protein in the elutes was analyzed through Sodium dodecyl sulfate polyacrylamide gel electrophoresis (SDS-PAGE) and western blotting using anti-His antibody.

### RNase and DNase Activity Assays

RNA degradation assays were used to examine whether the recombinant GmPru ar 1 protein displayed RNase activity according to the method of Zubini et al. 2009 [48]. The samples containing 10 μg total soybean RNA were incubated with the presence of 10 μg Gly m 4l protein at 37°C for 0, 2 and 4 h in 50 μL of 50 mM buffer at different pHs (citrate for pH 3, MES for pH 5, phosphate for pH 7, and CHES (N-cyclohexyl-2-amino-ethanesulfonic acid) for pH 9). At pH 7, RNase activity of the recombinant Gly m 4l protein was also checked in the presence of zeatin. Samples containing 10 μg total soybean RNA were incubated with 10 μg recombinant Gly m 4l protein and 10 mM zeatin at 37°C for 30 min, 1, 2, 3 and 4 h. The samples incubated with the recombinant Gly m 4l protein alone or boiled Gly m 4l protein and zeatin were used as the negative controls. The result was examined by 1% agarose gel electrophoresis (AGE) then visualized under UV light.

Meanwhile, the RNase activity of the recombinant Gly m 4l protein on yeast tRNA (Sigma, USA) was also assayed by determining the generation of acid-soluble, UV-absorbing species as described by Wang and Ng [64]. Briefly, 10 μg recombinant Gly m 4l protein was incubated with 100 μg tRNA in 75 μL of phosphate buffer (pH 7.0) at 37°C for 30 min, and the reaction was terminated by introduction of 12 μL of 3.4% ice-cold perchloric acid, then the samples were centrifuged at 1,500×g for 15 min at 4°C. The OD_260_ of the supernatant was read after appropriate dilution. One unit of RNase activity is defined as the amount of enzyme that brings about an increase in OD_260_ of one per minute in the acid-soluble fraction per milliliter of reaction mixture. Negative controls were performed using buffer alone or the boiled recombinant Gly m 4l protein.

The DNase activity assay was performed following the methods as described by Zubini et al. [48] and He et al. [65] with some modifications. Briefly, 10 μg total DNA prepared from soybean leaves was incubated with 10 μg recombinant Gly m 4l protein in 50 μL reaction mixtures in the presence of 50 mM buffer under different pH conditions incubated for 4 h at 37°C. Negative control was performed using buffer alone for 4 h at 37°C. RNase and DNase activity assays were performed on three technical replicates.

### *In Vitro* Antimicrobial Activity of the Recombinant *Gly m 4l* Protein

The antimicrobial activity of the recombinant Gly m 4l protein was assayed by the hyphal growth inhibition method as described by Schlumbaum et al. [66] with some modifications. The pathogen *P*. *sojae* had been germinated on V8 juice agar plates at 25°C for 72 h until the colonies reached a diameter of 3.5 cm, and sterile filter paper discs of 0.6 cm diameter were placed at 1 cm from the growing front, followed by the application of 15 or 25 μg of the recombinant Gly m 4l protein onto the discs. After incubation at 25°C for 24 h, the pathogen growth zones were measured. Negative controls were performed using 25 μg of boiled Gly m 4l protein or buffer alone.

The *P*. *sojae* zoospores were developed according to the procedure of Ward et al. [62] with minor modifications. Ten colonies with a diameter of 0.8 cm of *P*. *sojae* were removed and suspended in 10 mL sterile water to develop zoospores. After the fourth replacement of water, 100 μg of the recombinant Gly m 4l protein was mixed with 10 mL of the supernatant and incubated at 25°C in the dark. After incubation in the dark for 16 h, the zoospore release was observed using a Leica DMI 6000 microscope (Wetzlar, Germany) and the concentration was estimated using a hemacytometer. Negative controls were performed using 100 μg of boiled recombinant Gly m 4l protein or buffer alone. Antimicrobial activity assay of the recombinant Gly m 4l protein was performed on three technical replicates.

### Vector Construction and Soybean Transformation

To over-express *Gly m 4l* gene under the cauliflower mosaic virus (CaMV) 35S promoter of pCAMBIA3301 vector (www.camia.org) containing the *bar* gene as the selective marker, the full-length coding region of *Gly m 4l* was cloned in frame into the pCAMBIA3301 vector with the primer pairs *Gly m 4l*-oF and *Gly m 4l*-oR (S1 Table). The recombinant construct 35S: *Gly m 4l* was introduced into *Agrobacterium tumefaciens* strain LBA4404 using the freeze-thaw method as described by Holsters et al. [67]. The *Agrobacterium*-mediated soybean transformation [68] was performed using cotyledonary nodes of ‘Dongnong 50’ soybean as explants. After darkness culture, shoot proliferation, elongation induction, root differentiation and plantlet regeneration, the regeneration plants were transferred into pots and grown in the greenhouse.

### Pathogen Response Assays of Transgenic Soybean Plants

The seeds of the T_0_ transgenic soybean plants were collected, dried at 25°C, and grown on soil. The T_1_ transgenic soybean plants were identified using PCR amplification with the primer pairs *bar*-F and *bar*-R to amplify regions of the *bar* reporter gene (see S1 Table for primer sequences). Then, the T_1_ transgenic soybean plants were further identified using Southern hybridization with the DIG High Prime DNA Labeling and Detection Starter Kit II (Roche Cat., Germany), and the probe used for Southern hybridization was a nucleotides of 403 bp from *bar* gene.

The fully expanded leaves of T_2_ transgenic soybean plants, of which the T_1_ plants were tested through PCR and Southern hybridization, were further identified with the primer pairs *Gly m 4l*-qF or *Gly m 4l*-qR using qPCR (see S1 Table for primer sequences) and performed to screen on resistance to *P*. *sojae* according to the procedure of Kim et al. [69] with minor modifications. Briefly, the living leaves inoculated with *P*. *sojae* were covered with a polytene bag to maintain the relative humidity and the soybean seedlings were incubated in a mist chamber at 25°C, with 90 ± 10% relative humidity under a 16 h photoperiod at a light intensity of 350 mol m^-2^ s^-1^ for investigation. After 48 h and 96 h, the disease symptoms on each leaf sample were observed and photographed using Canon IXUS 860IS camera. To further investigate the responses of *Gly m 4l-*overexpressing plants to *P*. *sojae* ingress, the seedlings of T_3_ transgenic soybean plants were identified with the primer pairs *Gly m 4l*-qF or *Gly m 4l*-qR using qPCR (see S1 Table for primer sequences), and the relative biomass of *P*. *sojae* in infected cotyledons of the selected T_3_ transgenic plants at the first-node stage (V1) [61] were assessed after 48 h incubation with zoospores suspension of *P*. *sojae*. The *P*. *sojae* zoospores were developed according to the procedure of Ward et al. [62] with minor modifications and the concentration was 8 × 10^5^ mL^-1^ estimated using a hemacytometer. The assessment of biomass of *P*. *sojae* was based on the transcript level of *P*. *sojae TEF1* (GenBank accession no. EU079791) in reference to soybean *EF1β* according to the method of Chacón et al. [70] (see S1 Table for *TEF1* and *EF1β* primer sequences). The pathogen response assays were performed on three biological replicates with their respective three technical replicates.

## Results

### Isolation and Sequence Analysis of *Gly m 4l*

**Fig 1:**
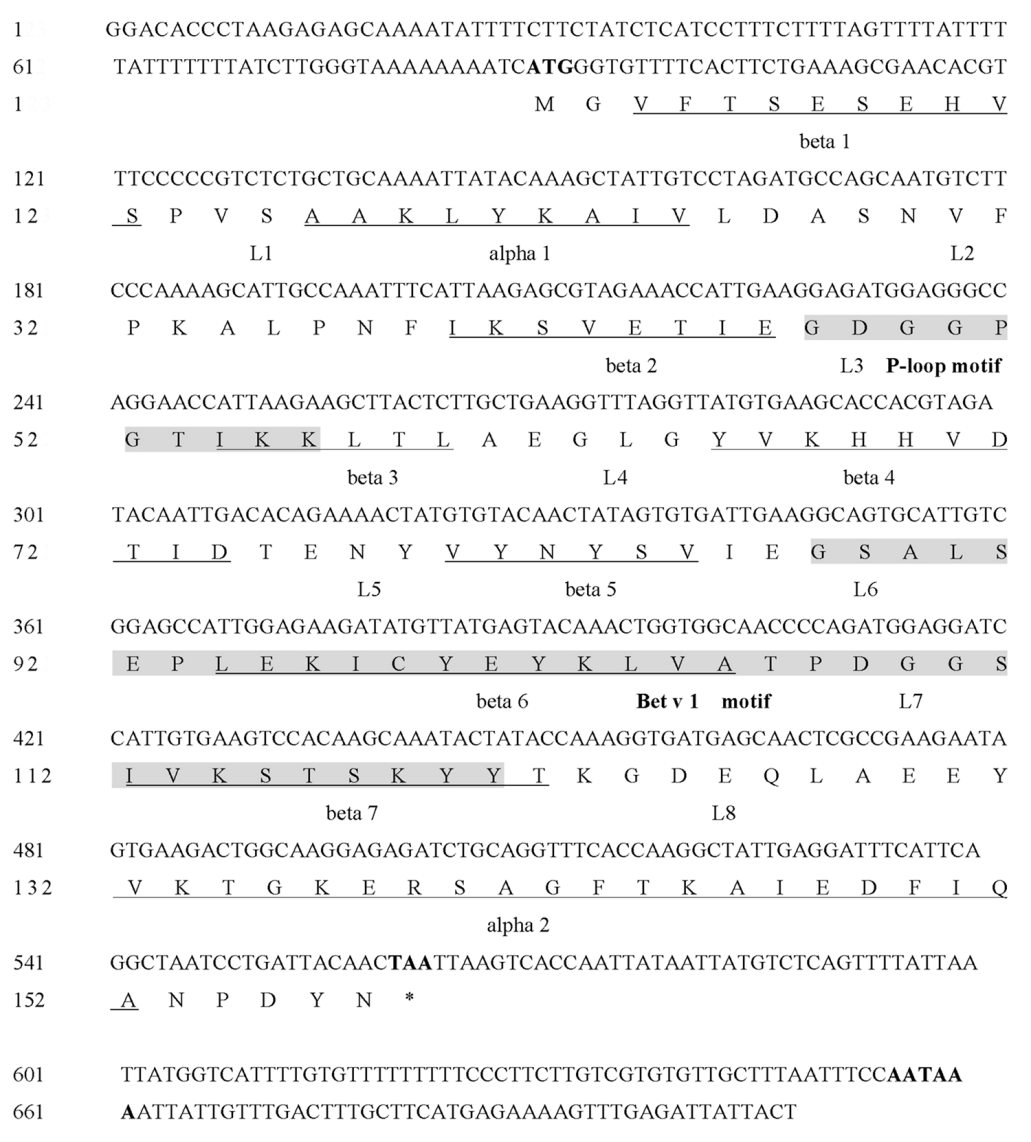
Nucleotide and amino acid sequence of *Gly m 4l*. The P-loop motif and Bet v 1 motif are shown in shadow. The α-helices and β-sheets are marked underlined.

**Fig 2:**
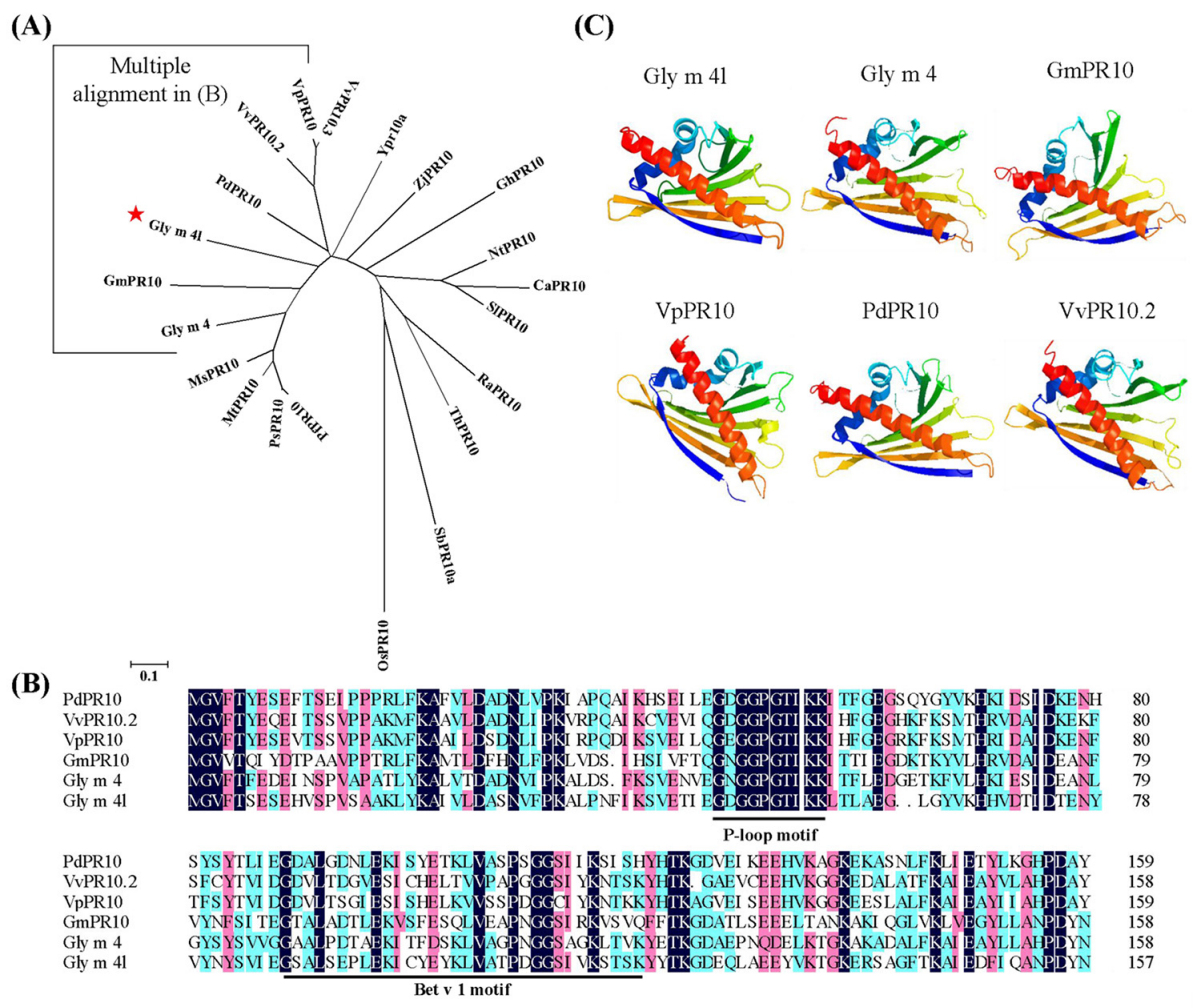
Characterization analysis of Gly m 4l. (A) Phylogeny analysis of Gly m 4l with 20 other PR10 proteins. The GenBank Accession numbers are as follows: PfPR10 (AAB07447.1), PsPR10 (AAA90954.1), MtPR10 (AES65085.1), MsPR10 (CAC37691.1), GmPR10 (XP_006582821.1), Gly m 4 (CAA42646.1), VvPR10.2 (CAC16165.1), VvPR10.3 (CBJ49381.1), Ypr10a (CAB94733.1), ZjPR10 (AGL07712.1), PdPR10 (ABW99634.1), VpPR10 (ABC86747.1), GhPR10 (AAG18454.1), NtPR10 (AEY11296.1), CaPR10 (ABC74798.1), SlPR10 (AHC08074.1), RaPR10 (ACH63224.1), ThPR10 (ACK38253.1), SbPR10a (AAW83207.1), and OsPR10 (BAD03969.1). (B) Alignment of amino acid sequences of Gly m 4l and the nearby 5 PR10 proteins. The ‘P-loop’ motif and the Bet v 1 motifs are marked underlined. (C) The tertiary structure of Gly m 4l protein and the comparison with that of the other five PR10 proteins.

The full-length cDNA sequence was obtained from ‘Suinong 10’ soybean using RACE. Alignment and phylogenetic tree analysis of the full-length amino acids sequence with the eight soybean allergen families reported showed identities of 53.46% homology with Gly m 4 protein, representing the highest homology (S1 Fig), and it was designated Gly m 4-like (Gly m 4l, GenBank accession no. HQ913577.1). As shown in Fig 1, the 707 bp *Gly m 4l* gene contains a single open reading frame (ORF) of 474 nucleotides encoding a polypeptide of 157 amino acids, with a calculated molecular mass of 17.14 kDa and a theoretical PI of 4.98. The nucleotide sequence showed a 5’ untranslated region (5’ UTR) of 88 nucleotides and a 3’ UTR of 145 nucleotides, along with a poly A signal (AATAAA) at 656–661 bp. The genomic *Gly m 4l* DNA sequence was also amplified, showing that *Gly m 4l* had an intron of 341 bps at 184 bp. The results of the database search revealed that Gly m 4l contained no signal peptide. The predicted structure of Gly m 4l protein included a conserved ‘P-loop’ (phosphate-binding loop) motif (GXGGXGXXK at residues 47–55 aa) and a Bet v 1 domain comprising 34 amino acids (at residues 87–120 aa), present in many PR 10 proteins. A neighbor-joining (NJ) phylogram was used to construct a phylogenetic tree based on the deduced sequence of Gly m 4l that contained other members of the PR10 family (Fig 2A), indicating that these proteins might share a common ancestor and display similar functions. The homology analysis of Gly m 4l with five nearby PR10 protein sequences showed that it shared 56.9% identity with PdPR10 (*Prunus domestica*) (ABW99634.1), representing the highest homology (Fig 2B). The prediction of the three dimensional (3D) structure of Gly m 4l based on the data from Phyre (http://www.sbg.bio.jc.ac.uk/phyre/), showed that this protein was very similar to that of the other PR10 proteins (Fig 2C). Gly m 4l contained a 24-amino-acid long α-helix (α2) at the C-terminus and a short α-helix (α1) at the N- terminus. The α2 was surrounded by seven anti-parallel β-sheets (β1 to β7) and the α1 was located between the β1 and β2 strands. The short loop structures were named L1 to L8 located between the α-helix and β- sheet (Fig 2A). The results suggested that Gly m 4l belonged to PR 10 protein family.

### The Transcript Abundance Patterns of *Gly m 4l*

**Fig 3:**
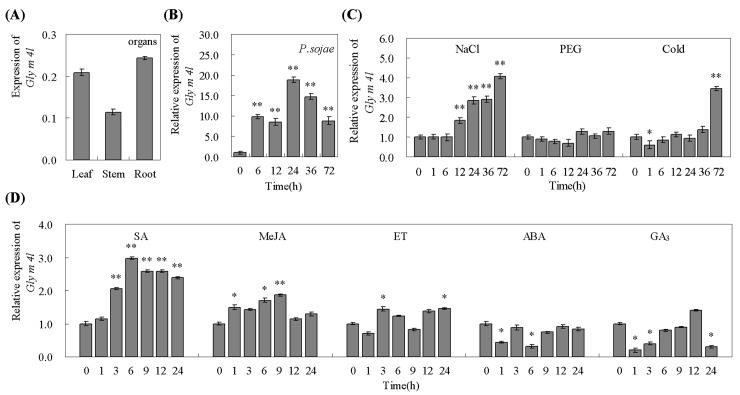
Expression patterns analysis of *Gly m 4l* by quantitative real-time PCR. (A) The transcript abundance of *Gly m 4l* in the root, stem and leaf. The Ct value of each sample was normalized to the Ct value of *GmEF1B*. (B) The transcript abundance of *Gly m 4l* in response to *P*. *sojae* infection. The Ct value of each sample was normalized to the Ct value of *GmActin4*, and the relative expression of *Gly m 4l* was compared with mock plants at the same time point. (C) and (D), The transcript abundance of *Gly m 4l* in response to various stresses. Exogenous chemicals are SA (0.5 mM), MeJA (100 μM), ET (0.2 mM ethephon), ABA (50 mM), GA_3_ (50 mg.L^-1^), NaCl (100 mM), PEG (20%), and cold (4°C). The Ct value of each sample was normalized to the Ct value of *GmActin4* and the relative expression of *Gly m 4l* was compared with mock plants at the same time point. The experiments were performed on three biological replicates with their respective three technical replicates and statistically analysed using Student’s t-test (*P<0.05, **P<0.01). Bars indicate standard error of the mean (SE).

Quantitative real-time PCR was performed to assess the tissue-specific expression of *Gly m 4l* in ‘Suinong 10’ soybean. The results showed that *Gly m 4l* was constitutively and highly expressed in the roots, followed by the leaves and stems (Fig 3A). The transcript abundance of *Gly m 4l* was investigated in the fully expanded unifoliolate leaves of ‘Suinong 10’ soybean in response to biotic and abiotic stresses. For *P*. *sojae* infection, a significant induction of *Gly m 4l* was detected from 6–72 h after the treatment, and the transcripts reached a maximum level at 24 h (Fig 3B). The transcript abundance of *Gly m 4l* under abiotic stresses was also investigated, such as NaCl, PEG, and cold (Fig 3C). NaCl treatment induced a significant up-regulation of *Gly m 4l* transcripts from 12–72 h. Cold induced an upregulation of *Gly m 4l* expression significantly only at 72 h after the treatment. There was little change for *Gly m 4l* transcript under the PEG stress. Meanwhile, the transcript abundance of *Gly m 4l* in response to exogenous chemicals including SA, MeJA, ET, ABA or GA_3_ was then carried out (Fig 3D). Sprayed with SA, the transcript of *Gly m 4l* was induced significantly from 3–24 h after the treatment, and reached a maximum level at 6 h. The *Gly m 4l* transcripts were also induced by MeJA and ET. There was almost down-regulation of the *Gly m 4l* transcript with the treatments of ABA and GA_3_.

### Subcellular Localization of *Gly m 4l* Protein

**Fig 4:**
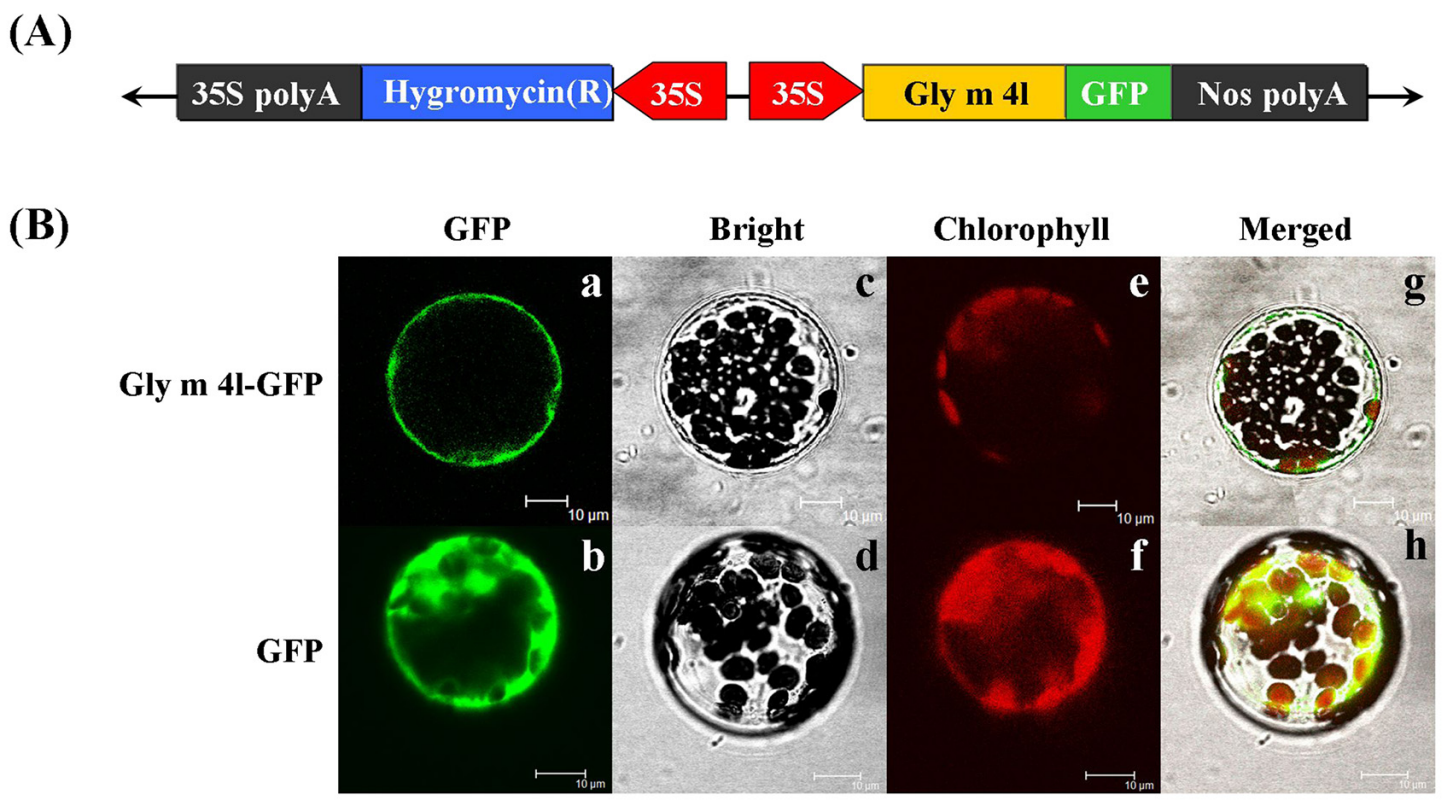
Subcellular localization analysis of *Gly m 4l*-GFP protein in Arabidopsis protoplasts. (A) Flow chart of construction 35S: Gly m 4l-GFP for subcellular localization analysis. (B) Images visualized by a Confocal Laser Scanning Microscopy. The images of bright-field (a and b), the GFP fluorescence (green) only (c and d), the chlorophyll autofluorescence (red) only (e and f) and combined ones (g and h) are shown. All scale bars indicate 10 μm.

To test the subcellular localization, the Gly m 4l protein was fused to the GFP coding sequences under the control of the CaMV 35S promoter (Fig 4A). Then, the newly constructed vector 35S: Gly m 4l-GFP was introduced into Arabidopsis protoplasts cells using PEG-mediated transient expression. As shown in Fig 4B, confocal microscopic observations showed that GFP was dispersed throughout the entire cells bombarded with the control plasmid 35S: GFP and the fusion Gly m 4l-GFP protein was localized exclusively to cell membrane, indicating that Gly m 4l was a cell membrane-localized protein.

### Purification of the Recombinant *Gly m 4l* Protein

**Fig 5:**
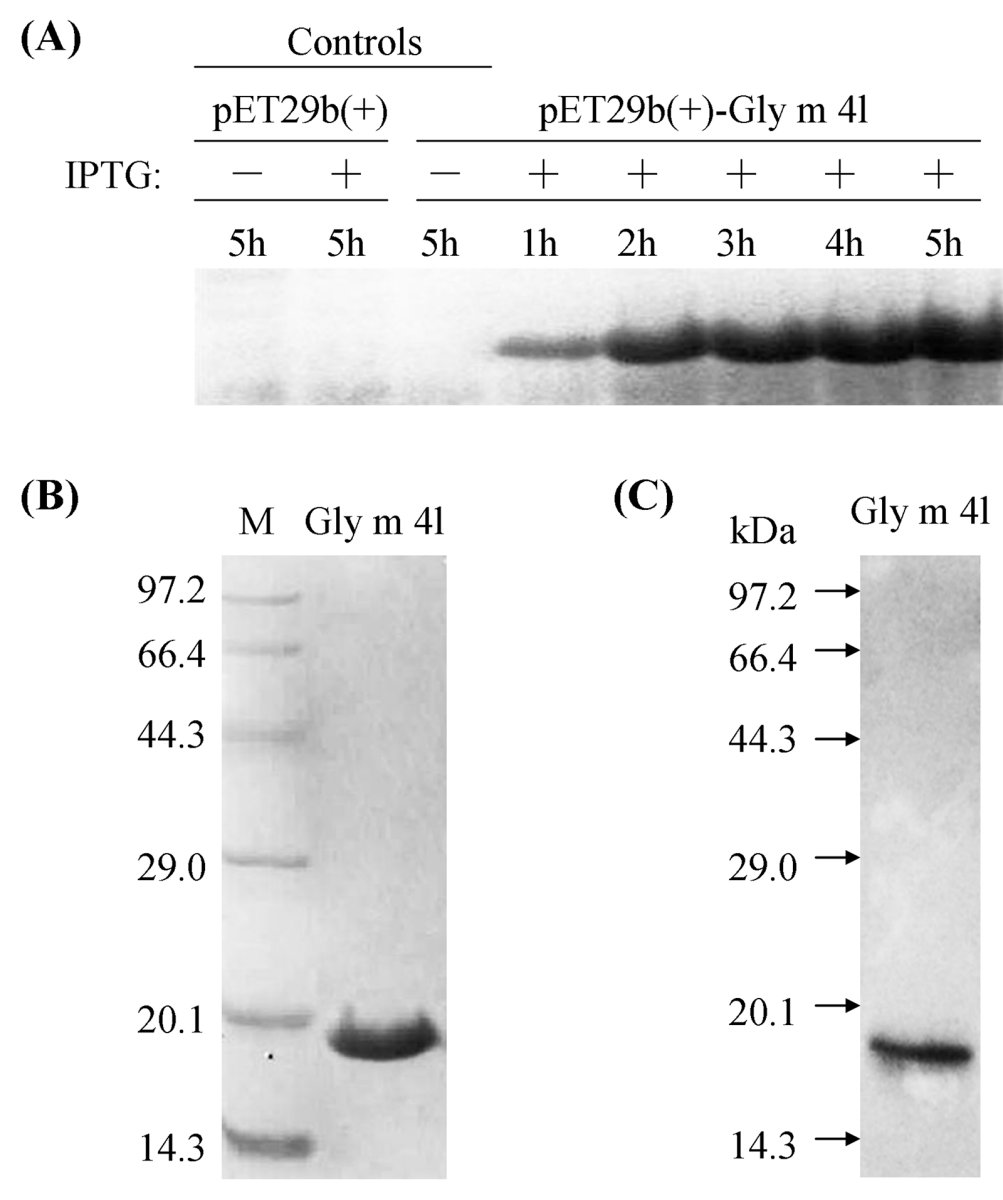
Analysis of the purified recombinant *Gly m 4l* protein. (A) The recombinant Gly m 4l protein induced with 0.5 mM IPTG at 37°C for 1–5 h in *E*.*coli* BL21 (DE3). (B) SDS-PAGE analysis of the purified recombinant Gly m 4l protein using His-Bind Kits. (C) The purified recombinant Gly m 4l protein analyzed by immunoblotting using anti-His antibody.

The expression of the recombinant Gly m 4l protein was remarkably enhanced after induction with 0.5 mM IPTG at 37°C for 1–5 h, reaching the maximum expression at 5 h, and the recombinant Gly m 4l protein was not detected in the control groups (Fig 5A). The recombinant Gly m 4l protein was purified using His-Bind Kits (EMD Millipore, USA), and the molecular weight of the purified protein was about 17 kDa in SDS-PAGE, consistent with the calculated molecular mass (17.14 kDa) (Fig 5B). Meanwhile, the Gly m 4l protein was also immunoprecipitated using anti-His antibody, and a single band appeared on the X-ray film in the regular western blotting (Fig 5C).

### RNase and DNase Activities of *Gly m 4l* Protein

**Fig 6:**
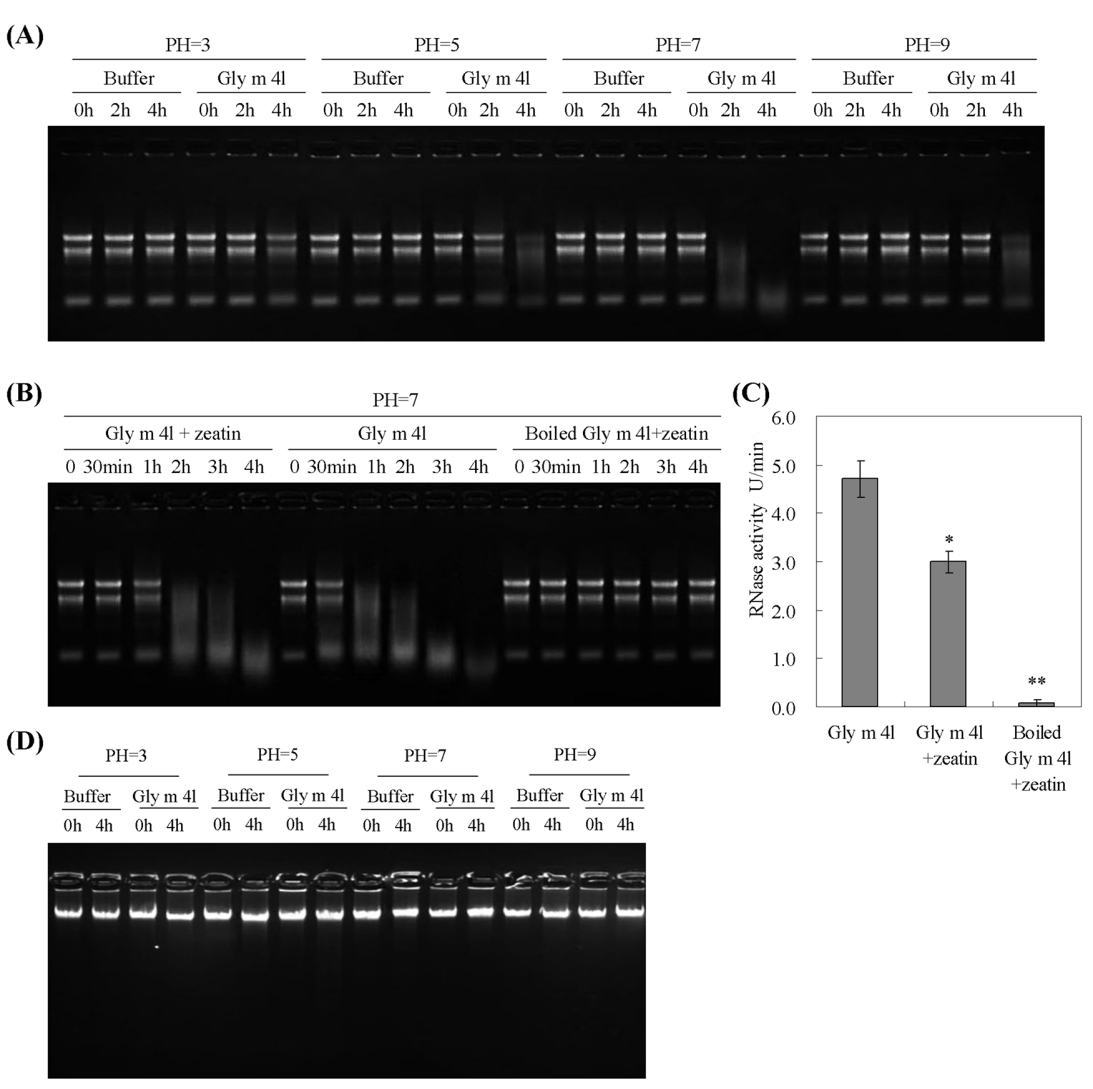
RNase and DNase activities of the recombinant *Gly m 4l* protein. (A) RNase activity of Gly m 4l protein under different pH conditions. Samples containing 10 μg total soybean RNA were incubated with the presence of 10 μg Gly m 4l protein at 37°C for 0, 2 and 4 h at pH 3, 5, 7 and 9 from left to right. (B) RNase activity of the recombinant Gly m 4l protein analyzed in the presence of zeatin using total soybean RNA at pH 7. Samples containing 10 μg total soybean RNA were incubated with the presence of 10 μg Gly m 4l protein and 10 mM zeatin at 37°C for 30 min, 1, 2, 3 and 4 h. The samples incubated with the presence of the recombinant Gly m 4l protein or boiled Gly m 4l protein and zeatin were used as negative controls. (C) RNase activity of the recombinant Gly m 4l protein analyzed in the presence of zeatin using yeast tRNA. Samples containing 100 μg yeast tRNA were incubated with the presence of 10 μg recombinant Gly m 4l protein and 10 mM zeatin at 37°C for 30 min. The samples incubated with the presence of Gly m 4l protein alone or boiled Gly m 4l protein and zeatin were used as controls. The results were calculated by the 2^−ΔΔCt^ method. (D) DNase activity of the recombinant Gly m 4l protein under different pH conditions. Samples containing 10 μg total soybean DNA and 10 μg Gly m 4l protein were incubated at 37°C for 0 and 4 h at pH 3, 5, 7 and 9 from left to right. The experiments were performed on three technical replicates and statistically analysed using Student’s t-test (*P<0.05, **P<0.01). Bars indicate standard error of the mean (SE).

To examine the RNase activity of the recombinant Gly m 4l protein, 10 μg total RNA from ‘Suinong 10’ soybean was incubated with 10 μg purified Gly m 4l protein at different pH conditions. As shown in Fig 6A, the RNase activities of Gly m 4l protein increased at 2 h and 4 h with the increase of pH from 3 to 7. At pH 9, almost no RNA digestion was detected at 2 h, while RNA was almost totally degraded at 4 h. To evaluate the effect of cytokinin on the RNase activity of the Gly m 4l protein, we examined the RNase activity in the presence of 10 mM zeatin at pH 7. The RNA digestion was delayed by 30 min in the presence of zeatin compared to that without zeatin (Fig 6B). Meanwhile, RNase activity of Gly m 4l was also checked using yeast tRNA, and the results showed that RNase activity of Gly m 4l towards yeast tRNA with the presence of zeatin was significantly lower than that without zeatin (Fig 6C).

Furthermore, the DNase activity of the Gly m 4l protein was also examined, and no hydrolysis was tested after 4 h of incubation at different pH conditions (Fig 6D).

### *In Vitro* Antimicrobial Activity of the Recombinant *Gly m 4l* Protein

**Fig 7:**
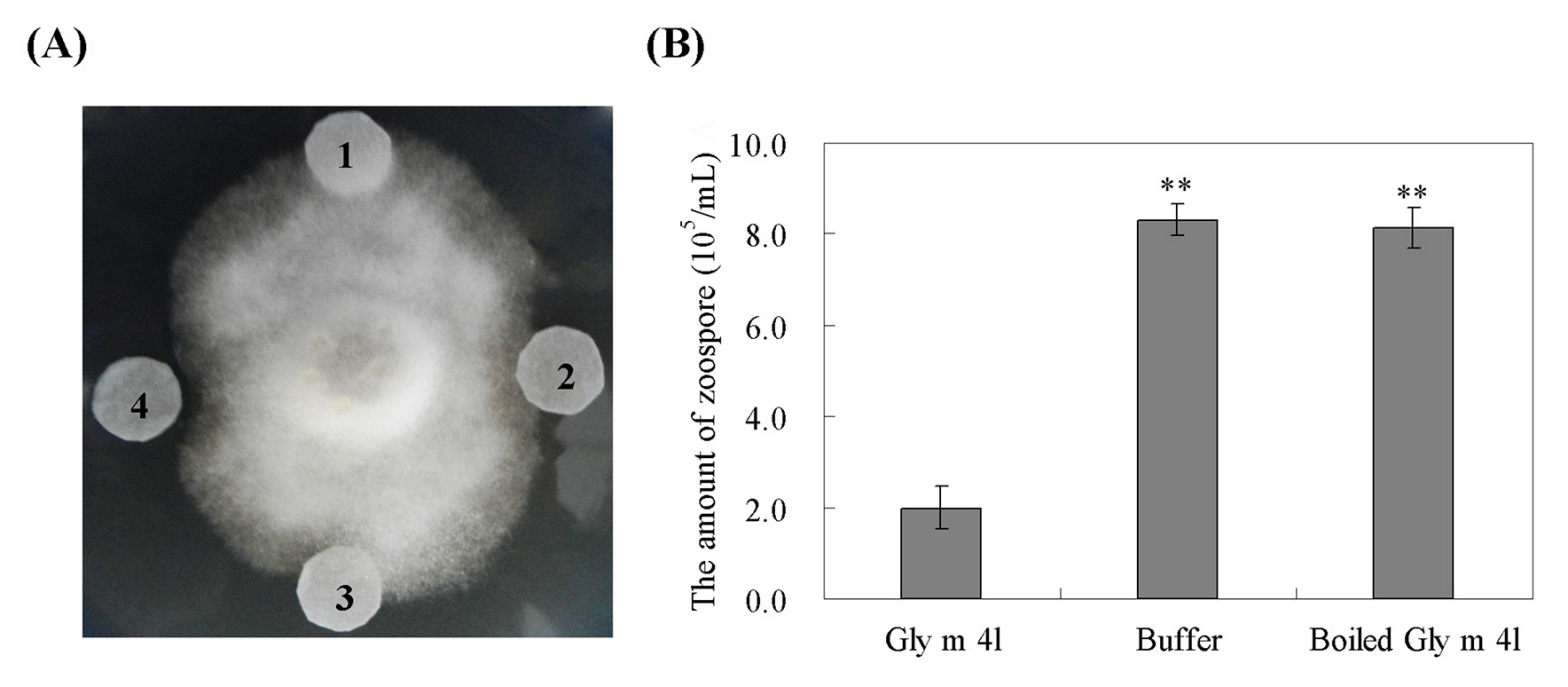
Antimicrobial activity of the recombinant *Gly m 4l* protein. (A) The antimicrobial activity of the recombinant Gly m 4l protein against the hyphal growth of *P*. *sojae*. 1, 25 μg boiled Gly m 4l protein. 2, 15 μg Gly m 4l protein; 3, Elution buffer. 4, 25 μg Gly m 4l protein. (B) The antimicrobial activity of the recombinant Gly m 4l protein against zoospore release of *P*. *sojae*. The experiments were performed on three technical replicates and statistically analysed using Student’s t-test (*P<0.05, **P<0.01). Bars indicate standard error of the mean (SE).

*In vitro*, inhibition activity of the recombinant Gly m 4l protein against hyphal growth of *P*. *sojae* was assayed, and hyphal growth was monitored from 24 to 72 h. After incubation for 72 h, 2–3 mm and 4–6 mm zones with inhibited hyphal growth were detected with 15 μg and 25 μg of the recombinant protein, respectively (Fig 7A). No inhibition of the growth of *P*. *sojae* was observed using discs containing elution buffer or boiled protein (Fig 7A). Antimicrobial activity of the recombinant Gly m 4l protein against *P*. *sojae* zoospores was also assayed. The amount of zoospores was significantly decreased with the presence of 100 μg recombinant Gly m 4l protein, compared to that with the presence of 100 μg boiled Gly m 4l protein or buffer alone (Fig 7B). These results suggested that the recombinant Gly m 4l protein possessed antibiotic activity against *P*. *sojae* through the inhibition of hyphal growth and zoospore release.

### Over-Expression of *Gly m 4l* in Soybean Enhances Resistance to *P*. *sojae*

**Fig 8:**
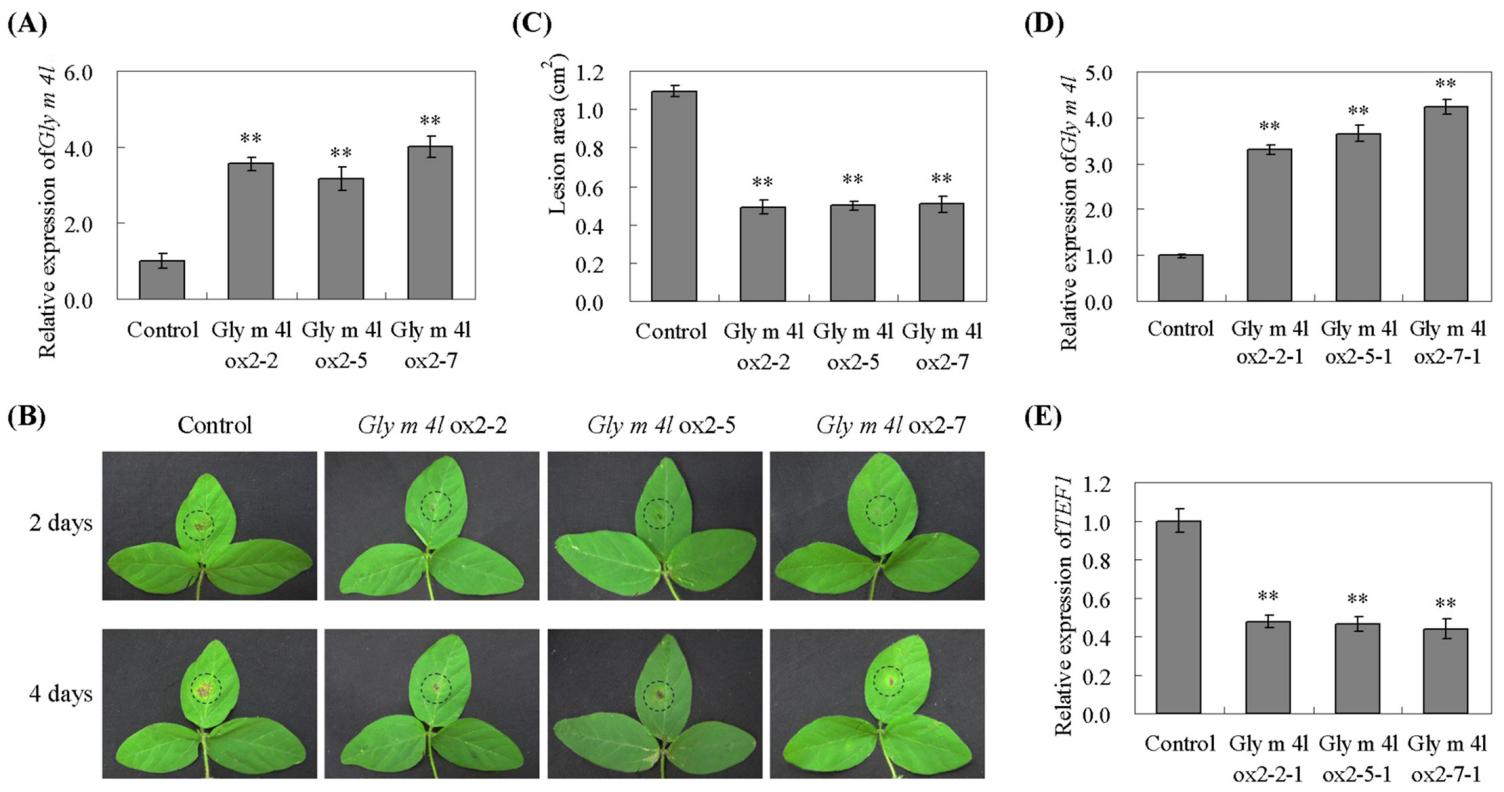
Response of *Gly m 4l* transgenic soybean plants to *P*. *sojae*. (A) Quantitative real-time PCR of the T_2_ transgenic soybean plants. (B) Disease symptoms on the leaves of the transgenic lines and non-transgenic lines treated with a *P*. *sojae* race 1 inoculum at 48 h and 96 h. (C) The lesion area of the transgenic lines and non-transgenic lines were detected after 96 h of incubation with *P*. *sojae*. (D) Quantitative real-time PCR of the T_3_ transgenic soybean plants. (E) Quantitative real-time PCR analysis of *P*. *sojae* relative biomass based on the transcript level of the *P*. *sojae TEF1* gene. The experiment was performed on three biological replicates with their respective three technical replicates and statistically analysed using Student’s t-test (*P<0.05, **P<0.01). Bars indicate standard error of the mean (SE).

To investigate whether over-expression of *Gly m 4l* enhances resistance in transgenic plants, the T_1_ transgenic soybean plants, confirmed through PCR and Southern hybridization (S2 Fig), were developed to T_2_ transgenic soybean plants and selected by qPCR (Fig 8A) to assay the pathogen response (S3 Fig). After 96 h incubation with *P*. *sojae*, the leaves of the non-transgenic soybean plants exhibited clear and large lesions compared to those of the transgenic plants (Fig 8B), and the lesion area of the transgenic soybean lines is significantly (P<0.01) smaller than that of non-transgenic soybean plants after 96 h incubation with *P*. *sojae* (Fig 8C). Moreover, the T_3_ transgenic soybean plants were confirmed by qPCR (Fig 8D), and the relative biomass of *P*. *sojae* in infected cotyledons after 48 h incubation with zoospores suspension of *P*. *sojae* was also analyzed. The results indicated that the biomass of *P*. *sojae* based on the transcript level of the *P*. *sojae TEF1* gene was significantly (P<0.01) lower in *Gly m 4l*-overexpressing transgenic plants than that in non-transgenic ones (Fig 8E). These results indicated that the over-expression of *Gly m 4l* in soybean plants improved resistance to *P*. *sojae*.

## Discussion

Until recently, a variety of allergens have been identified into eight families in soybean (http://www.allergen.org), namely Gly m 1, Gly m 2, Gly m 3, Gly m 4, Gly m 5, Gly m 6, Gly m 7 and Gly m 8. Gly m 4 is a member of the superfamily of PR10 proteins [53–55], but there is almost no study on the disease resistance of this protein. Although soybean PR10 genes were first identified in 1992 [71], there is limited information concerning the potential functions of these proteins *in vitro* and *in vivo*. In a previous study, we functionally characterized *GmPR10* (GenBank accession no. FJ960440) in soybean [14]. In the present study, we provide the first description of Gly m 4l in soybean (*Glycine max* [L.] Merr.), showing identities of 53.46% homology with Gly m 4 protein, a member of the PR10 family, containing a Bet v1-motif and P-loop motif. We also isolated and characterized the PR10 member Gly m 4l in response to pathogen (*P*. *sojae*) infection in soybean plants.The amino acid sequence, structure, subcellular location and other biological functions of PR10 proteins indicated that the major members of the PR10 family can be grouped into two different classes: intracellular pathogenesis-related proteins (IPR) with homology to ribonucleases [1] and (S)-norcoclaurine synthases (NCS) [72]. The sequence analysis indicated that Gly m 4l contained no signal peptide and the subcellular localization analysis showed that Gly m 4l protein was localized at cell membrane, suggesting that Gly m 4l is an intracellular protein similar to IPRs of the PR10 family. Gly m 4l also shared several conserved features of known IPR PR10 proteins, such as a small molecular mass, acidic pI and an intron of 76–359 nucleotides [8, 73]. Most *PR10* genes are clustered in chromosomes [8, 14], and the analysis of *Gly m 4l* based on data obtained from the internet (http://soybase.org/GlycineBlastPages/) indicated that a total of nine genes were clustered into nine linkage groups, namely Gm 01, 07, 08, 09, 10, 14, 15, 17, and 18.

In this study, our results indicated that mRNA transcripts of *Gly m 4l* were remarkably increased by SA stress, but relatively low under MeJA and ET treatments, and almost decreased with ABA and GA_3_ treatments (Fig 3). Therefore, we speculated that *Gly m 4l* might play a key role in soybean plants resistance to *P*. *sojae* mainly depending on SA signaling, which is one of the important component of signal transduction and could activate plant defense responses against pathogen attack [74]. In addition, the expression of *Gly m 4l* was also induced by NaCl and cold. It is likely that high-salinity and low-temperature resulted in increased cytosolic Ca^2+^ [75–76], and Ca^2+^ was the second messenger for hypersensitive response induction or defense gene expression and leads to up-regulation of PR proteins [77].

Some of PR10 proteins have previously been shown to possess RNase or DNase activity and performed a link to plant defense strategy repertoire, such as JIOsPR10 from *Oryza sativa* [39], VpPR10.2 from *Vitis pseudoreticulata* [65], JcPR10a from *Jatropha curcas* [78], VpPR10.1 from *Vitis pseudoreticulata* [16], VpPR10.4 and VpPR10.7 from *Vitis pseudoreticulata* [79]. On the basis of these findings, it supports the hypothesis that RNase activity was potentially crucial for plant defense, such as activating the death of infected cells to limit pathogen invasion, regulating and controlling mRNA transcription upon pathogen infection or other stresses, and directly degrading pathogenic RNA [40, 48]. Previous reports showed that a limited number of proteins have been targeted to non-classical secretory pathway without an N-terminal signal peptide [80]. Although Gly m 4l contained no signal peptide (Fig 1) and the subcellular localization analysis showed that Gly m 4l protein was localized at cell membrane (Fig 4), whether or not Gly m 4l is a secretory protein is unclear yet. Under *P*. *sojae* stress, one possibility is that Gly m 4l protein in soybean could be targeted to non-classical secretory pathway and secreted into the intercellular space, then it performed antimicrobial function by directly degrading pathogenic RNA to inhibit hyphal growth or zoospore release. Another is that Gly m 4l protein could not be targeted to non-classical secretory pathway, then it performed antimicrobial functional role through degrading RNA of haustorium, which is formed and stick into cytoplasm of susceptible soybean with *P*. *sojae* infection [81], to limit pathogen invasion.

Members of PR10 family have been reported that they have antimicrobial activities in transgenic plants, as examples, *VpPR10*.*2* gene from *Vitis pseudoreticulata* responded to *Plasmopara viticola* infection and over-expression of *VpPR10*.*2* in the host plant enhanced resistance to *Plasmopara viticola* [65], the over-expression of *PgPR10-2* and *PgPR10-4* conferred a tolerance against fungal infection [69, 82], and over-expression of *GmPR10* in soybean enhanced resistance to *P*. *sojae* [14]. In the present study, *Gly m 4l* over-expression transgenic soybean plants enhanced resistance to *P*. *sojae in vivo* and showed a lower biomass of *P*. *sojae* than that in non-transgenic soybean plants, suggesting that Gly m 4l could inhibit *P*. *sojae* infection and might be a useful tool for managing Phytophthora root and stem rot in soybean plants.

## Conclusions

In conclusion, a new member of the PR10 protein family, Gly m 4l, was isolated from resistant soybean ‘Suinong 10’, showed significant increased transcript abundance with *P*. *sojae* inoculum, and also induced by SA, NaCl, MeJA and ET. The recombinant Gly m 4l protein showed RNase activity and displayed directly antimicrobial activity that inhibited hyphal growth and reduction zoospore release in *P*. *sojae*. Further analyses showed that the RNase activity of the recombinant Gly m 4l protein on tRNA was significantly inhibited in the presence of zeatin. Over-expression of *Gly m 4l* in susceptible ‘Dongnong 50’ soybean showed enhanced resistance to *P*. *sojae*. These results indicated that the Gly m 4l protein played an important role in the defense of soybean against *P*. *sojae* infection.

## Supporting Information

S1 Fig Alignment and phylogenetic tree analysis of the full-length amino acids sequence with other soybean allergens. (TIF)

S2 Fig Southern blot analysis of transgenic soybean plants. control, non-trangenic soybean plant; ox2 and ox7, T_1_ indepently trangenic soybean plants; 3301, pCAMBIA3301 vector.
(TIF)

S3 Fig Disease symptoms on the leaves of the transgenic lines and non-transgenic lines treated with a *P*. *sojae* race 1 inoculum. (TIF)

S1 Table Oligonucleotide primers used in this study. (DOC)
