# Supplementary figures and images for: A Novel Pathogenesis-Related Class 10 Protein Gly m 4l, Increases Resistance upon Phytophthora sojae Infection in Soybean (Glycine max [L.] Merr.)
Source: PLoS One. 2015 Oct 16;10(10):e0140364. doi: 10.1371/journal.pone.0140364 (PMC4608668; doi:10.1371/journal.pone.0140364)

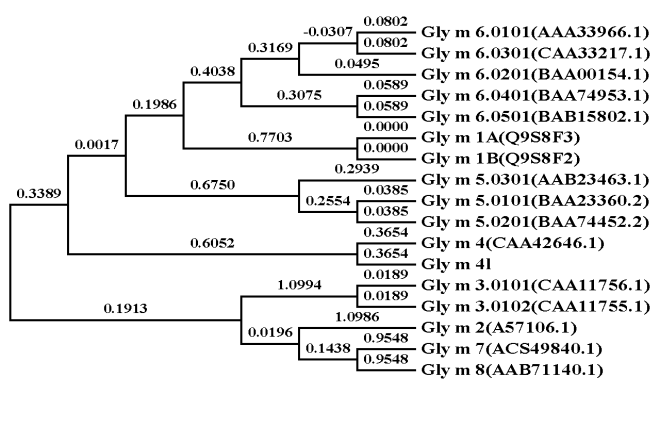

Supplement: S1 Fig — (TIF) [file pone.0140364.s001.tif]

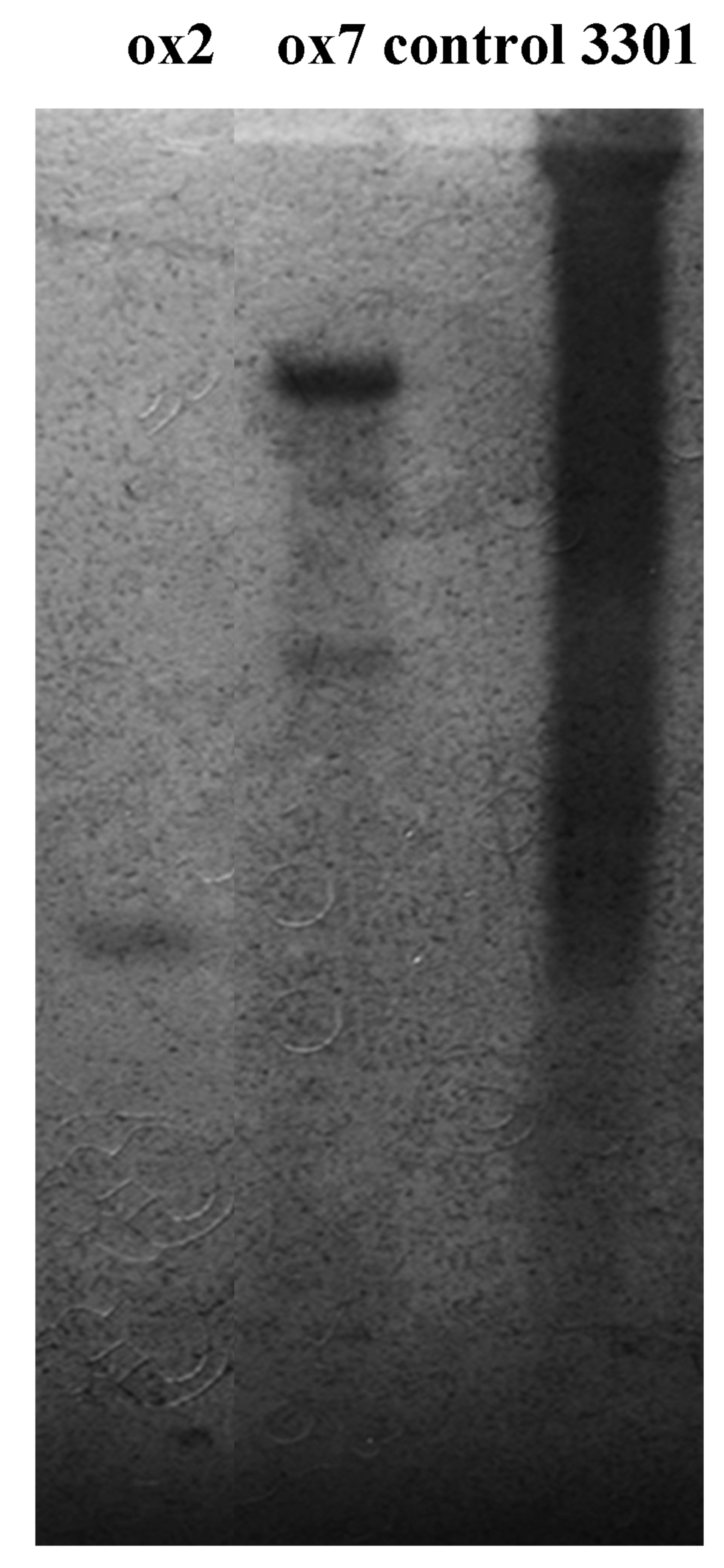

Supplement: S2 Fig — control, non-trangenic soybean plant; ox2 and ox7, T1 indepently trangenic soybean plants; 3301, pCAMBIA3301 vector. (TIF) [file pone.0140364.s002.tif]

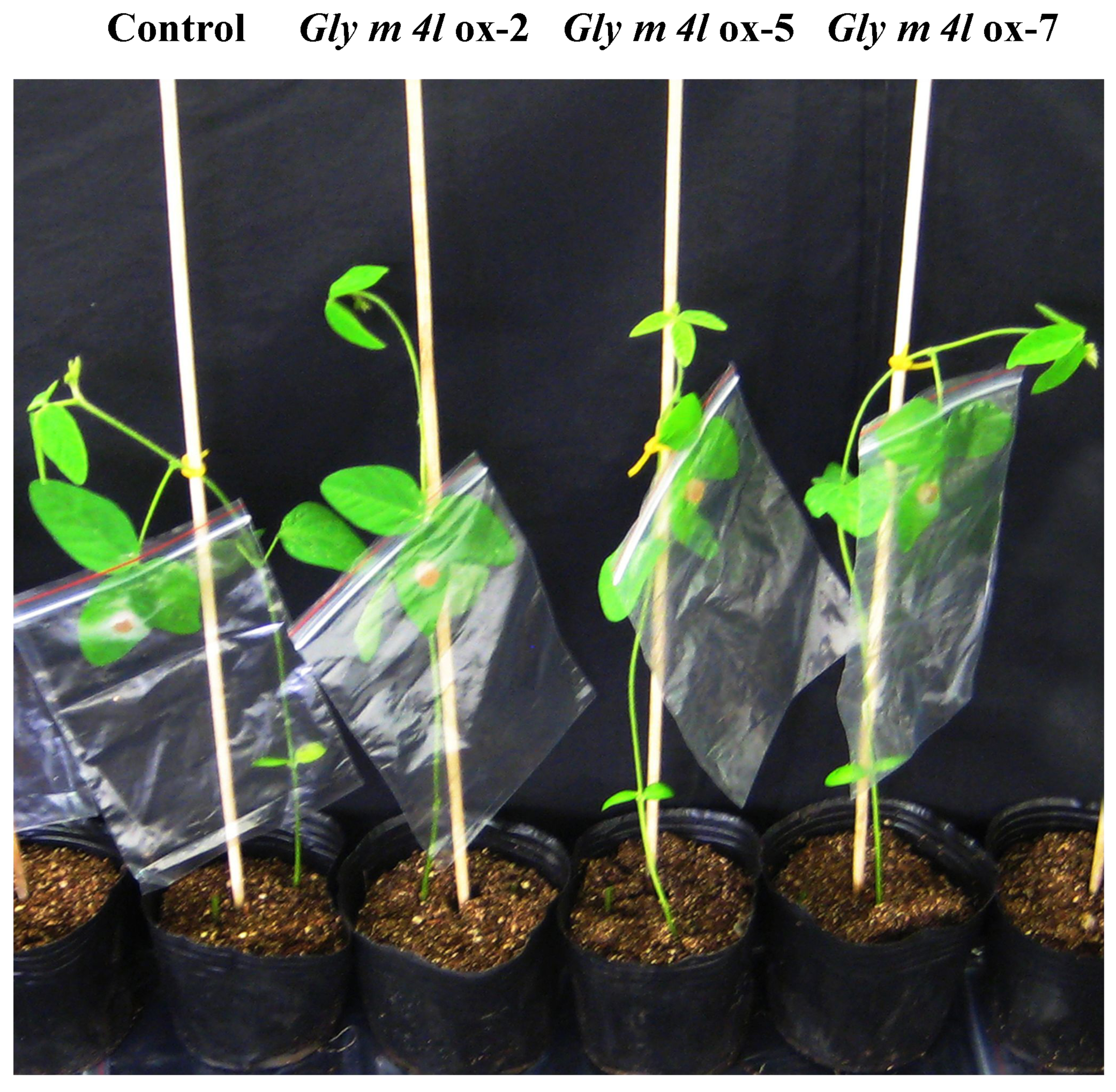

Supplement: S3 Fig — (TIF) [file pone.0140364.s003.tif]
