# Supplementary material for: A Novel Pathogenesis-Related Class 10 Protein Gly m 4l, Increases Resistance upon Phytophthora sojae Infection in Soybean (Glycine max [L.] Merr.)
Source: PLoS One. 2015 Oct 16;10(10):e0140364. doi: 10.1371/journal.pone.0140364 (PMC4608668; doi:10.1371/journal.pone.0140364)
Supplement: S1 Table — (DOC) [file pone.0140364.s004.doc]

S1 Table. Oligonucleotide primers used in this study.

|  | Name | Sequences |
| --- | --- | --- |
| Gene cloning | *GSP1* | CTCAATAGCCTTGGTGAAACCTG |
|  | *GSP2* | CGAGTTGCTCATCACCTTTGG |
|  | F | GCTCTAGAGGACACCCTAAGAGAGCAAA |
|  | R | CGAGCTCAAAGCAACACACGACAAGAA |
| qPCR | *Gly m 4l*-qF | GCCGATGTACAAACTGGTGGCAA |
|  | *Gly m 4l*-qR | CCTCAATAGCCTTGGTGAAACCTGC |
|  | *GmEF1β*-F | GACCTTCTTCGTTTCTCGCA |
|  | *GmEF1β*-R | CGAACCTCTCAATCACACGC |
|  | *GmActin4*-F | GATCTACCATGTTCCCAAGT |
|  | *GmActin4*-R | ATAGAGCCACCAATCCAGAC |
|  | *TEF1*-F | TGATCGTGCTGAACCACCC |
|  | *TEF1*-R | CGAGCGACGGTCCATCTT |
| GFP | *Gly m 4l-*lF | CGAGCTCATGGGTGTTTTCACTTCTGA |
|  | *Gly m 4l-*lR | GACTAGTAAAGCAACACACGACAAGAA |
| Over-expression | *Gly m 4l*-oF | CAGATCTCATGGGTGTTTTCACTTCTGA |
|  | *Gly m 4l*-oR | GGGTAACCAAAGCAACACACGACAAGAA |
|  | *bar*-F | ATATCCGAGCGCCTCGTGCAT |
|  | *bar*-R | GGTCTGCACCATCGTCAACCACT |
